# Connection of spectral pattern of carbohydrate molecular structure to alteration of nutritional properties of coffee by-products after fermentation

**DOI:** 10.5713/ab.24.0021

**Published:** 2024-04-26

**Authors:** Samadi, Xin Feng, Luciana Prates, Siti Wajizah, Zulfahrizal, Agus Arip Munawar, Weixian Zhang, Peiqiang Yu

**Affiliations:** 1 Animal Husbandry Department, Agricultural Faculty, Universitas Syiah Kuala, Darussalam-Banda Aceh, 23111, Indonesia; 2 Research Centre for Innovation and Feed Technology, Universitas Syiah Kuala, Banda Aceh 23111, Indonesia; 3 Department of Animal and Poultry Science, College of Agriculture and Bioresources, University of Saskatchewan, 51 Campus Drive, Saskatoon, SK, S7N 5A8, Canada; 4 Department of Agricultural Engineering Universitas Syiah Kuala, Darussalam-Banda Aceh 23111, Indonesia; 5 Henan University of Animal Husbandry and Economy, Zhengzhou, Henan 450046, China

**Keywords:** ATR-FTIR Molecular Spectroscopy, By-products from Coffee Processing, Carbohydrate, Fermentation, Molecular Structure, Nutrient Supply, Ruminant System

## Abstract

**Objective:**

The objective of this study was to determine internal structure spectral profile of by-products from coffee processing that were affected by added-microorganism fermentation duration in relation to truly absorbed feed nutrient supply in ruminant system.

**Methods:**

The by-products from coffee processing were fermented using commercial fermentation product, consisting of various microorganisms: for 0 (control), 7, 14, 21, and 28 days. In this study, carbohydrate-related spectral profiles of coffee by-products were correlated with their chemical and nutritional properties (chemical composition, total digestible nutrient, bioenergy values, carbohydrate sub-fractions and predicted degradation and digestion parameters as well as milk value of feed). The vibrational spectra of coffee by-products samples after fermentation for 0 (control), 7, 14, 21, and 28 days were determined using a JASCO FT/IR-4200 spectroscopy coupled with accessory of attenuated total reflectance (ATR). The molecular spectral analyses with univariate approach were conducted with the OMNIC 7.3 software.

**Results:**

Molecular spectral analysis parameters in fermented and non-fermented by-products from coffee processing included structural carbohydrate, cellulosic compounds, non-structural carbohydrates, lignin compound, CH-bending, structural carbohydrate peak1, structural carbohydrate peak2, structural carbohydrate peak3, hemicellulosic compound, non-structural carbohydrate peak1, non-structural carbohydrate peak2, non-structural carbohydrate peak3. The study results show that added-microorganism fermentation induced chemical and nutritional changes of coffee by-products including carbohydrate chemical composition profiles, bioenergy value, feed milk value, carbohydrate subfractions, estimated degradable and undegradable fractions in the rumen, and intestinal digested nutrient supply in ruminant system.

**Conclusion:**

In conclusion, carbohydrate nutrition value changes by added-microorganism fermentation duration were in an agreement with the change of their spectral profile in the coffee by-products. The studies show that the vibrational ATR-FT/IR spectroscopic technique could be applied as a rapid analytical tool to evaluate fermented by-products and connect with truly digestible carbohydrate supply in ruminant system.

## INTRODUCTION

Due to the anti-nutritional and anti-physiological factors, such as tannins and caffeine, direct use of coffee by-products for animal feed is not suitable [1]. The fermented coffee pulp total phenolic compounds concentrations are significantly decline compared to those which unfermented [2,3]. The fermentation process can detoxify the coffee pulp so it can be used as an alternative to animal feed. In previous study, we reported the fermentation induced protein structure changes associated with nutrient availability [4]. However, there was no investigation that determined the relationship between ruminant nutrition and internal structural alterations of carbohydrate in by-products from coffee processing caused by added microbe fermentation duration.

Published research reports [5–7] showed that evaluation of truly digestible nutrient supply to dairy cows from feeds could be predicted with feed structure spectral features. Yan et al [5] reported that the carbohydrate nutritional value of faba bean plant forage could be estimated by its structure spectral peak area and height intensities of chemical functional groups. Xin and Yu [6] also reported the similar findings. Further study by Refat et al [7] showed that vibrational spectroscopic technique with attenuated total reflectance (ATR)-Fourier transform infrared spectroscopy (FTIR) and chemometrics could be used as a fast and non-destructive tool to reveal internal association between structure and nutrition in barley silage. Peng et al [8] and Yu et al [9] also reported that the DRIFT, FTIR, ATR-FTIR and SR-IMS spectroscopic techniques enables to evaluate physicochemical features of feeds.

Yu et al [9] and Yan et al [5] indicated that molecular spectroscopy (eg. DRIFT, FTIR, ATR-FTIR, SR-IMS spectroscopy), unlike conventional wet chemical analysis methods which destroy internal structure during the processing, is able to reveal chemical and nutritional values from the inherent structures. As a result, the researchers apply FTIR to study interactive association between inherent molecular structure of feed, food and plant and nutritional, degradable and digestive characteristics, and to investigate the alteration or changes of structure during various treatment and processing [10]. However, research on the impact of microorganism fermentation duration of by-products from coffee processing to inherent molecular structure change is limited.

In this study, it was hypothesized that the carbohydrate-related spectral profiles in the by-products from coffee processing could be altered during microorganism fermentation and the alteration of structural spectral profiles could be related to carbohydrate nutritional characteristics of the fermented by-products. There could be some close relationship between the chemical composition, carbohydrate fractions and bioenergy profiles and unique internal spectral profiles of carbohydrates from the fermented by-products.

The objectives of this study were: i) to reveal changes in by-products from coffee processing fermented with different days with regard to carbohydrate-related chemical composition, bioenergy value, milk value of feed (FMV), carbohydrate-subfraction and internal spectral profiles of carbohydrates from the fermented by-products; ii) to determine alteration of carbohydrate inherent structure spectral characteristics of by-products during fermentation; iii) to investigate the internal association of nutritional parameters of fermented by-products in carbohydrates with its spectral features of chemical functional groups in fermented coffee by-products.

## MATERIALS AND METHODS

This study was approved by the University of Saskatchewan Animal Care Committee (USACC) with the Animal Use Protocol No. 19910012 and the animals used in this research were cared for and handled in compliance with the Canadian Council of Animal Care regulations [11]. Authors confirm that EU and Canadian standards for the protection of animals and/or feed legislation have been met.

### Fermentation study

Coffee from plantation by-products were gathered (wet processed), and they were subsequently dried in an oven at 60°C for 24 hours, until 90% of the DM was retained. Fermentation was carried out using the commercial fermentation product Saus Burger Pakan (SBP; 1%). SBP is made up of a variety of microorganisms (cellulolytic, lactic acid, amylolytic, proteolytic, and xylanolytic bacteria) and herbal extracts. SBP was activated with 3% molasses for 5 hours before being combined with coffee by-products. The samples were grinded and fermented with SBP for 0, 7, 14, 21, and 28 d. To optimize microbial fermentation, 3% sago was used as an energy source. Water was also added to the fermenting mix to achieve a water content of 40%. The fermentation experiment was carried out in anaerobic containers at room temperature (28°C to 30°C), with a total of 15 containers arranged, three for each time point.

The containers were opened at each fermentation time point, and the samples were dried in an air-ventilated oven (60°C) until consistent weight was achieved. Dried samples were ground using Retsch ZM-200 (Brinkmann Instruments Ltd., Mississauga, Canada) for chemical composition analysis (screen size: 1 mm) and spectral collection of chemical functional groups (screen size: 0.12 mm) at the SRP ministry strategic research lab with spectroscopy (Department of Animal and Poultry Science, University of Saskatchewan, Canada).

### Chemical analysis

The fermented by-product samples from coffee processing were determined for chemical compositions including dry matter (DM; AOAC 930.15), organic matter, crude protein (CP; AOAC 984.13), crude fat or ether extract (EE, AOAC 920.39), ash (AOAC 942.05), and starch (AOAC 996.11) following the procedures of Association of Official Analytical Chemists [12]. The published methods by Van Soest et al [13] were applied for feed fibre analyses including acid detergent lignin (ADL) and acid (ADF) and neutral (NDF) detergent fibers with Ankom A200 technique (Ankom Technology, Fairport, NY, USA). In the residues from fibre analysis (NDF, ADF), both acid (ADICP) and neutral (NDICP) detergent insoluble CP were determined. Soluble CP (SCP) in the fermented by-product samples was also determined Roe [14]. According to NRC (2001), total CHO (%) was calculated as 100–EE–CP–ash, non-fiber CHO (NFC, %) was calculated as 100–(NDF–NDICP)–EE–CP–ash, and hemicellulose (%) and cellulose (%) were calculated as NDF–ADF and ADF–ADL, respectively. The protein-related chemical profiles were reported previously. In this study, carbohydrate-related chemical profiles were used for interactive association study with carbohydrate molecular structure spectral profiles.

### Fractionation of carbohydrate fractions with CNCPS 6.5 system

The updated CNCPS 6.5 system [15] describes carbohydrate subfractions, including CA (CA1, volatile fatty acids; CA2, lactic acids; CA3, other organic acids; CA4, sugars); CB1 (rapid degradable fraction, starch); CB2 (intermediary degradable fraction, soluble fiber); CB3 (slowly degradable fraction, available cell wall) and CC (unavailable cell wall). Based on carbohydrate subfractions, the estimated degradable and undegradable carbohydrate supply as well as intestinal digestion of carbohydrate subfractions were evaluated for by-products from coffee processing affected by fermentation duration. The detailed parameters determined include i) rumen degradable (RD) carbohydrates including RDCA4, RDCB2, RDCB3 and total RDC; ii) rumen undegradable carbohydrates including RUCA4, RUCB2, RUCB3, RUCC, and total RUC; iii) intestinal digestion (ID) of subfractions including IDCA4, IDCB2 and IDCB3 [13,15].

### Bioenergy values and total digestible nutrients

Total digestible nutrients (TDN) and bioenergy values in the by-products from coffee processing affected by microorganism fermentation duration were determined using NRC method [15,16]. The studied parameters include total digestible CP (tdCP), NDF (tdNDF), NFC (tdNFC), and fatty acid (tdFA), TDN, digestive energy (DE_1x_, DE_p3x_), metabolizable energy (ME, ME_p3x_), net energy for maintenance (NE_m_), gain (NE_g_), and lactation (NE_L3x_) in fermented by-products.

### Molecular spectroscopy ATR-FTIR on carbohydrate structure

The dried fermented coffee by-products samples from 0, 7, 14, 21, and 28 d fermentation from 15 containers with three containers for each time point were scanned for FTIR spectra using JASCO FT/IR-4200 with sampling assessor of attenuated total reflection (ATR) (JASCO Corp., Tokyo, Japan) at the SRP Chair lab at University of Saskatchewan (Canada).

Each fermented and non-fermented by-product sample was scanned at mid-IR range (ca. 4,000 to 700 cm^−1^). For each sample, five repetitions were used to be collected for spectra. Each sample spectrum was scanned for 256 times. The resolution was set at 4 cm^−1^ for each spectrum. To offset background noise, the background spectrum from each sample was collected [5,17]. The previous studies [5,7,18–20] showed that carbohydrate-related spectral regions in plant-based biomaterials include total carbohydrate (at ca. 938 to 1,186 cm^−1^ region), cellulosic chemical compound (at ca. 1,186 to 1,289 cm^−1^ region) and structural carbohydrate (ca. 1,186 to 1,486 cm^−1^ region) [18,21]. Spectral peak heights and peak areas within each region were analyzed with OMNIC 7.3 (SpectraTech, Madison, WI, USA).

The detailed spectral parameters reported by previous researchers [5,7,18–20] included STCHO: structural carbohydrate (area at ca. 1,488 to 1,184 cm^−1^); CC: cellulosic compounds (area at ca. 1,300 to 1,184 cm^−1^; height at ca. 1,237 cm^−1^); NSHCO: non-structural carbohydrates (area at ca. 1,184 to 924 cm^−1^); lignin compound (height at ca. 1,507 cm^−1^); CH_bending (height at ca. 1,460 cm^−1^); STCHO_pk1: structural carbohydrate peak1 (1st peak height at ca. 1,418 cm^−1^); STCHO_pk2: structural carbohydrate peak2 (2nd peak height at ca. 1,397 cm^−1^); STCHO_pk3: structural carbohydrate peak3 (3rd peak height at ca. 1,320 cm^−1^); HC: hemicellulosic compound (height at ca. 1,150 cm^−1^); NSCHO_pk1: non-structural carbohydrate peak1 (1st peak height at ca. 1,102 cm^−1^); NSCHO_pk2: non-structural carbohydrate peak2 (2nd peak height at ca. 1,078 cm^−1^); NSCHO_pk3: non-structural carbohydrate peak3 (3rd peak height at ca. 1,030 cm^−1^) [5,7,19,20].

### Statistical analysis

The chemical, nutritional and spectral data of fermented by-products samples were determined using the MIXED procedure (SAS Institute, Inc., Cary, NC, USA) with fermentation time (different dates) as fixed effects and fermentation run as random effect. The residual analysis was performed to validate the model assumptions. The data normality check was determined using SAS univariate procedure with normal option. Orthogonal polynomial contrasts were used to evaluate linear and quadratic effects of fermentation duration (0, 7, 14, 21, 28) with equal time spacing treatment. The contrast between control (non-fermentation by products) and fermentation (average of fermentation for 7, 14, 21, and 28 days) was used to evaluate the overall difference between the fermentation and non-fermentation. The significance was declared at p<0.05 and the trend was declared at p< 0.10.

The correlation between spectral parameters and nutrition were performed using the SAS CORR procedure (SAS Institute, Inc., USA). The Spearman option was used because some of the data was not normally distributed. Carbohydrate molecular spectral features were used to associate with carbohydrate nutritional characteristics.

## RESULTS AND DISCUSSION

### Effect of fermentation duration on carbohydrate-related chemical profiles of by-products from coffee processing

Effect of microorganism fermentation duration on carbohydrate-related chemical profiles of by-products from coffee processing are showed in Table 1. Carbohydrate (CHO) content tended (p = 0.064) to linearly reduced with increasing microorganism fermentation duration from 0 to 28 days. The NFC content was also linearly reduced (p = 0.026) from 52% to 47% of DM. As to NDF and ADF, there was no linear and quadratic relationship (p>0.10) with microorganism fermentation duration. However, ADL was linearly reduced (p<0.01) from 1.34% to 1.20% of DM. The sugar content of by-products was quadratically reduced (p<0.01) with increasing fermentation duration. These results indicated that fermentation process with SBP had a dramatical impact on NFC, sugars, and lignin without significantly impacting on fibers (NDF and ADF). Yan et al [5] reported microorganism degradation in rumen changed total chemical composition contents not only structure carbohydrate but also non-structural carbohydrate in faba samples. Recently, Himmelsbach et al [22] and Yang et al [23] reported chemical profiles was altered after adding fibrolytic enzyme into whole plant faba bean silage for ruminants.

### Effect of fermentation duration on total digestible nutrient and energy profiles of by-products from coffee processing

The effect of microorganism fermentation length on TDN and energy profiles of by-products from coffee processing are presented in Table 2. Increasing fermentation duration results linearly decreased tdNDF (p = 0.009) and tended to linearly increase tdNFC (p = 0.079) without affecting tdCP. The tdFA content was quadratically increased from 0.93 to 1.22% of DM. Fermentation duration tended to have a quadratic impact (p = 0.069) on total TDN_1x_ content in coffee by-products. It reduced TDN_1x_ from 722 to 698 g/kg of DM. Yan et al [5] reported microorganism degradation in rumen changed digestible nutrient contents in faba samples. Yang et al [23] and Refat et al [7] also reported nutrient supply was altered after either adding fibrolytic enzyme or rumen microorganism degradation in faba silage and barley silage, respectively.

As to energy value of coffee by-products, microorganism fermentation duration had a quadratic impact (p<0.10) on all digestible energy (DE_1x_, DE_p3x_), metabolizable energy (ME, ME_p3x_), and net energy values (NE_L3x_, NE_m_, NE_g_). These results indicated that microorganism fermentation process resulted in quadratic decrease in TDN and digestible (DE_1x_, DE_p3x_), metabolizable (ME, ME_p3x_), and net energy values of fermented by-products from fermentation.

### Effect of fermentation duration on carbohydrate subfraction profiles, intestinal digestion and milk value of by-products from coffee processing

The results of carbohydrate partitioning fractions, the degradable and undegradable fractions, and intestinal digestion of by-products from coffee processing samples according to CNCPS 6.5 system are showed in Tables 3–5 respectively. Microorganism fermentation (Table 3) significantly reduced (p<0.01) CA4 fraction from 9.1 to 1.1 g/kg of CHO and increased (p = 0.05) CB2 fraction from 42.8 to 46.5 g/kg of CHO in a quadratic pattern (p<0.01) with fermentation duration (0 to 28 days). The CB3 fraction were linearly increased (p = 0.017) and The CC fraction was linearly decreased (p< 0.01) with increasing fermentation duration (Table 3). These results indicated that microorganism fermentation process resulted in a quadratic decrease in water-soluble carbohydrates of CA4 fraction and a quadratic increase in soluble fiber of CB2 fraction and a linear decrease in available fibre of CB3 fraction of coffee by-products.

The microorganism fermentation resulted in a quadratic decrease (p<0.01) in RDCA4 fraction from 5.29% to 0.64% of DM without significantly (both linearly and quadratically) impacting on RDB2 and RDCB3 (Table 4). Total RDC were linearly decreased (p = 0.028) from 45.79% to 42.81% of DM with increasing fermentation duration (Table 4). Fermentation also had quadratic decrease (p<0.01) RUCA4 fraction from 1.58% to 0.10% of DM without significantly linearly and quadratically impacting on RUB2 and RUCB3 (Table 4). Total RUC was slightly linearly decreased (p = 0.005) with increasing fermentation duration (Table 4). These results indicated that fermentation process resulted in quadratic decrease in rumen degradable water-soluble carbohydrates and linear decrease in total rumen degradable fraction of coffee by-products.

Fermentation had no significantly linearly and quadratically impact on IDCB2 and IDCB3 (Table 5). These results indicated that fermentation process did not affect intestinal digestion of soluble fiber (CB2) and available NDF (CB3) of coffee by-products. As to feed milk value, the microorganism fermentation (Table 6) tended to quadratically reduce (p = 0.055) FMV fraction from 2.27 to 2.19 kg/kg of by-products. Refat et al [7], Himmelsbach et al [22], and Yang et al [23,24] reported adding fibrinolytic enzymes impacted CNCPS fractions and supply from whole plant faba silage to dairy cows.

### Effect of fermentation duration on carbohydrate related molecular spectral profiles of by-products from coffee processing, revealed by vibrational molecular spectroscopy

The results of carbohydrate-related spectral profiles of by-products from coffee processing impacted by the microorganism fermentation duration are showed in Table 7. According to the results, several spectral parameters of fermented by-products were significantly or tended to be significantly linearly affected (p<0.01) by fermentation duration in terms of CC peak area at ca. 1,300 to 1,184 cm^−1^ (p = 0.054) with an increase from 4.36 to 4.67 AU, lignin compounds peak height at ca. 1,507 cm^−1^ (lignin height, p = 0.089) with an increase from 0.001 to 0.007 AU, STCHO_pk3 height at ca. 1,320 cm^−1^ (p = 0.027) with an increase from 0.123 to 0.135 AU, CC height at ca. 1,237 cm^−1^ (p = 0.092) with an increase from 0.076 to 0.080 AU, and HC height at ca. 1,150 cm^−1^ (p = 0.041) with a decrease from 0.131 to 0.115 AU.

However, fermentation had no significant linear and quadratic impact (p>0.10) on STCHO peak area at ca. 1,488 to 1,184 cm^−1^ and NTCHO NTCHO peak area at ca. 1,184 to 924 cm^−1^. The fermentation duration also had no significantly linear and quadratic impact (p>0.10) on STCHO_pk1 height at ca. 1,418 cm^−1^) and STCHO_pk2 height at ca. 1,397 cm^−1^, NSCHO_pk1 height at ca. 1,102 cm^−1^, NSCHO_pk2 height at ca. 1,078 cm^−1^, and NSCHO_pk3 height at ca. 1,030 cm^−1^.

These results indicated that fermentation process only affected specific spectral groups of functional chemicals and linearly increased ATR-FTIR spectral peak area and height intensity of cellulosic chemical compounds, spectral peak intensity height of lignin chemical compounds, spectral peak3 height of structural carbohydrate, and linearly decreased spectral peak height of hemicellulose chemical compounds in fermented coffee processing by-products.

Yan et al [5] reported that when rumen incubation increased, ATR-FTIR spectral peak intensity was reduced with exception of the 1st spectral peak intensity height of total CHO, peak intensity height of cellulosic chemical compound, spectral peak intensity area of cellulosic chemical compound as well as the 4th spectral peak intensity height of structural CHO. The alteration was relatively small [5]. However, Xin and Yu [6] also found that rumen incubation time were found to have significant effect to all spectral parameters. In this study, carbohydrate spectral intensity was increased with increasing fermentation time for only several spectral profiles with exception of STCHO_pk1 and STCHO_pk2 height and NSCHO_pk1, NSCHO_pk2, and NSCHO_pk3 height.

Xin and Yu [6] reported that after rumen incubation, the ATR-FTIR peak spectral intensity of the ruminal incubation residues were dramatically lower than spectral intensity from original feeds in comparison with our results from fermented by-product from coffee processing in this study and the reported results from whole plant faba bean forage by Yan et al [5]. Yan et al [5] also indicated that sometimes univariate molecular spectral analysis fails to discriminate the subtle spectral difference or spectral change between samples and treatments based on unique spectral intensity parameters while multivariate spectral analysis which utilize whole spectral absorption information can achieve. Comparison among three studies ([5,6] and current study), the results are inconsistent, which indicates the sophisticate impacts by rumen microbial degradation and enzymic digestion on the ATR-FTIR spectral intensity patterns. This is mainly due to the internal complicated structural make-up difference between the samples in three studies.

### Revealing correlation-ship between feed quality and nutritional profiles and ATR-FTIR spectral intensity parameters and profiles of by-products from coffee processing

Therefore, the relationship with correlation analysis was conducted for by-products from coffee processing impacted by SBP microorganism fermentation duration is presented in Tables 8 and 9. Yan et al [5] demonstrated that during rumen fermentation and degradation, the alteration of ATR-FTIR spectral peak area and height intensity parameters significantly reflects nutrient digestion and degradation kinetics by ruminal microorganism. As for CHO chemical profiles (Table 8), CHO-related ATR-FTIR spectral peak intensity parameters of coffee processing by-products affected by SBP microorganism fermentation duration had significant correlations with ADF fibre content, especially with structural CHO’s 2nd spectral intensity peak height (r = −0.60) and total CHO’s 3rd spectral intensity peak height (r = −0.53), ADL content with the structural CHO’s 1st peak height (r = −0.80), and starch content with the ATR-FTIR spectral intensity peak height of lignin (r = −0.64). Yan et al [5] reported that for faba bean plant composition samples, ATR-FTIR spectral intensity peak parameters had stronger correlation with faba bean plant starch content, especially faba bean plant structural CHO’s ATR-FTIR spectral intensity peak area (r = −0.88), structural CHO’s 2nd spectral peak height (r = −0.92) and total CHO’s 3rd peak height (r = −0.95).

The TDN (Table 9), CHO-related spectral intensity parameters of fermented coffee by-products samples have significant correlation with TND_1x_ content, especially structural CHO’s ATR-FTIR 2nd peak height (r = 0.66) and structural CHO’s ATR-FTIR 3rd peak height (r = 0.62) and cellulosic chemical compound’s ATR-FTIR spectral intensity peak area (r = −0.62).

As for energy values (Table 9), carbohydrate-related spectral profiles of fermented coffee by-products samples have significant correlation with digestible, metabolizable and net energy content, especially peak area of total carbohydrate (r = 0.52 to 0.66), total CHO’s ATR-FTIR 1st spectral intensity peak height (r = 0.51 to 0.59), total CHO’s ATR-FTIR 2nd spectral intensity peak height (r = 0.66 to 0.74), total CHO’s ATR-FTIR 3rd spectral intensity peak height (r = 0.60 to 0.62) and cellulosic chemical compound’s spectral intensity peak area (r = −0.60 to 0.62). In the published correlation study, Yan et al [5] reported that there was a significantly strong correlation between ruminal degradation kinetics (degradation rate, potential degradable fraction, effective degradable NDF, unavailable CC fraction) and ATR-FTIR spectral peak intensity parameters of ruminal residues in faba bean plant composition samples.

The current and previous studies indicate that the ATR-FTIR spectral peak intensity changes and alteration during microorganism fermentation or ruminal digestion may be related or associated to the cleaving of bond during microbial fermentation and degradation and hence interfere digestion of cell wall by enzyme and microorganisms [5,25].

## CONCLUSION

In conclusion, alteration of CHO nutrition value by microorganism fermentation duration with adding SBP additive in the coffee processing by-products was reflected in the alteration of its ATR-FTIR spectral peak intensity features. The results show that ATR-FTIR spectroscopic technique could be applied as a rapid evaluation method to determine fermented by-products CHO nutrient supply in complicated ruminant systems. Future study is needed to use unique ATR-FTIR spectral intensity parameters and/or whole or partial spectral ATR-FTIR wavenumber range of fermented coffee processing by-products to develop nutrient supply evaluation and prediction equations using multivariate molecular spectral analyses with chemometrics in complicated ruminant system.

## Figures and Tables

**Table 1:** Effect of fermentation duration on carbohydrate profile of by-products from coffee bean processing

Carbohydrate profile (% DM)	Control (C)	Fermentation duration (days) (F)				SEM	p-value	Contrast^1)^		
		
	F0	F7	F14	F21	F28			C vs F	L	Q
CHO	75.27	73.52	74.21	74.44	73.72	0.332	0.028	0.005	0.064	0.233
NFC	51.99	46.17	49.43	49.48	47.21	0.758	0.002	<0.001	0.026	0.199
NDF	37.49	39.56	37.52	37.60	38.90	0.436	0.019	0.092	0.539	0.728
ADF	28.88	30.96	28.41	29.10	29.15	0.438	0.018	0.307	0.361	0.620
ADL	1.34	1.46	1.20	1.19	1.20	0.015	<0.001	0.001	<0.001	0.628
Starch	1.54	1.39	1.29	1.70	1.39	0.192	0.602	0.655	0.989	0.793
Sugar	6.88	0.83	2.56	2.18	1.29	0.181	<0.001	<0.001	<0.001	<0.001

DM, dry matter; SEM, standard error of mean; CHO, total carbohydrate; NFC, non-fibre carbohydrate; NDF, neutral detergent fibre; ADF, acid detergent fibre; ADL, acid detergent lignin.

1) Polynomial contrast: L, linear; Q, quadratic.

**Table 2:** Effect of fermentation duration on digestible nutrients and predicted energy value of by-products from coffee bean processing

Item	Control (C)	Fermentation duration (days) (F)				SEM	p-value	Contrast^1)^		
		
	F0	F7	F14	F21	F28			C vs F	L	Q
Digestible nutrients (% DM)										
tdNDF	21.56	22.81	22.35	22.29	22.93	0.220	0.010	0.001	0.009	0.342
tdNFC	42.18	38.29	40.40	40.74	39.09	0.606	0.009	0.003	0.079	0.263
tdCP	13.34	14.11	13.88	13.38	13.93	0.196	0.066	0.053	0.501	0.361
tdFA	0.93	0.71	0.99	1.04	1.22	0.070	0.006	0.433	0.002	0.060
Total digestible nutrient (g/kg DM)										
TDN_1x_^2)^	721.9	698.5	718.8	717.7	717.1	0.32	0.002	0.031	0.361	0.069
Predicted energy value^3)^ (Mcal/kg DM)										
DE_p3x_	2.95	2.87	2.94	2.93	2.93	0.010	0.001	0.035	0.236	0.053
ME_p3x_	2.52	2.44	2.52	2.52	2.51	0.010	0.001	0.036	0.229	0.057
NE_L3x_	1.58	1.53	1.58	1.57	1.58	0.007	0.001	0.037	0.218	0.055
ME	2.63	2.56	2.62	2.62	2.62	0.009	0.001	0.040	0.231	0.060
NE_m_	1.72	1.66	1.71	1.71	1.71	0.008	0.001	0.039	0.225	0.058
NE_g_	1.10	1.05	1.10	1.09	1.09	0.007	0.001	0.037	0.222	0.056

SEM, standard error of mean; DM, dry matter; tdNDF, truly digestible neutral detergent fibre; tdNFC, truly digestible non-fibre carbohydrate; tdCP, truly digestible crude protein; tdFA, truly digestible fatty acid.

1) Polynomial contrast: L, linear; Q, quadratic.

2) TDN_1x_, total digestible nutrient at maintenance level (NRC, 2001).

3) DE_p3x_, digestible energy at production level at 3 times maintenance intake; ME_p3x_, metabolizable energy at production level at 3 times maintenance intake; NE_L3x_, net energy for lactation at production level at 3 times maintenance intake; ME, metabolizable energy; NE_m_, net energy for maintenance; NE_g_, net energy for gain.

**Table 3:** Effect of fermentation duration on carbohydrate subfractions of by-products from coffee bean processing, partitioned according to the newly updated CNCPS 6.5

Item^1)^	Control (C)	Fermentation duration (day) (F)				SEM	p-value	Contrast^2)^		
		
	F0	F7	F14	F21	F28			C vs F	L	Q
CA4 (% CHO)	9.14	1.13	2.94	3.44	1.75	0.237	<0.001	<0.001	<0.001	<0.001
CB2 (% CHO)	42.84	45.04	46.48	46.03	45.46	0.886	0.106	0.014	0.051	0.050
CB3 (% CHO)	40.72	44.13	42.68	42.43	44.05	0.550	0.007	0.001	0.017	0.274
CA4 (% DM)	6.88	0.83	2.18	2.56	1.29	0.181	<0.001	<0.001	<0.001	<0.001
CB2 (% DM)	32.24	33.12	34.51	34.27	33.51	0.737	0.268	0.079	0.145	0.107
CB3 (% DM)	31.37	33.38	31.97	31.88	32.82	0.337	0.011	0.012	0.221	0.530
CC (% DM)	3.22	3.50	2.89	2.87	2.89	0.036	<0.001	<0.001	<0.001	0.635

SEM, standard error of mean; DM, dry matter; NDF, neutral detergent fibers.

1) Carbohydrates (CHO) partition in to the subfractions: CA4, water-soluble carbohydrates (sugars); CB1, carbohydrate fraction intermediately degradable (starch); CB2, soluble fiber; CB3, available NDF; CC, unavailable NDF, which were partitioned according CNCPS v6.5.

2) Polynomial contrast: L, linear; Q, quadratic.

**Table 4:** Effect of fermentation duration on rumen degradable and undegradable content of carbohydrate subfractions in by-products from coffee bean processing, estimated according to updated CNCPS 6.5

Item^1)^ (% DM)	Control (C) F0	Fermented (F)				SEM	p-value	Contrast^2)^		
	
		F7	F14	F21	F28				L	Q
RDCA4	5.29	0.64	1.67	1.97	0.99	0.139	<0.001	<0.001	<0.001	<0.001
RDCB2	24.80	25.48	26.54	26.36	25.78	0.567	0.268	0.079	0.145	0.107
RDCB3	15.68	16.69	15.98	15.94	16.41	0.168	0.011	0.012	0.221	0.503
Total RDC	45.79	42.81	44.21	44.28	43.19	0.462	0.008	0.001	0.028	0.189
RUCA4	1.58	0.19	0.50	0.59	0.29	0.041	<0.001	<0.001	<0.001	<0.001
RUCB2	7.44	7.64	7.96	7.90	7.73	0.170	0.268	0.079	0.145	0.107
RUCB3	15.68	16.69	15.98	15.94	16.41	0.168	0.011	0.012	0.221	0.530
RUCC	3.22	3.50	2.89	2.87	2.89	0.036	<0.001	<0.001	<0.001	0.635
Total RUC	27.95	28.03	27.35	27.31	27.33	0.174	0.028	0.048	0.005	0.452

DM, dry matter; SEM, standard error of mean.

1) RDC, predicted rumen degradable carbohydrates; RUC, rumen undegradable carbohydrates partitioned according CNCPS v6.5.

2) Polynomial contrast: L, linear; Q, quadratic.

**Table 5:** Estimated intestinal digestion of carbohydrate subfractions by using CNCPS in coffee beans submitted to different fermentation time

Item^1)^ (% DM)	Control (C)	Fermentation duration (day) (F)				SEM	p-value	Contrast^2)^		
		
	F0	F7	F14	F21	F28				L	Q
IDCA4	1.58	0.19	0.50	0.59	0.29	0.041	<0.001	<0.001	<0.001	<0.001
IDCB2	5.95	6.11	6.37	6.32	6.18	0.136	0.268	0.079	0.145	0.107
IDCB3	12.55	13.35	12.79	12.75	13.13	0.134	0.011	0.012	0.221	0.530
IDCC	20.09	19.66	19.66	19.67	19.61	0.132	0.137	0.014	0.050	0.159

DM, dry matter; SEM, standard error of mean; NDF, neutral detergent fibers.

1) Carbohydrates (CHO) partition in to the subfractions: CA4, water-soluble carbohydrates (sugars); CB2, soluble fiber; CB3, available NDF; CC, unavailable NDF, which were partitioned according CNCPS v6.5.

2) Polynomial contrast: L, linear; Q, quadratic.

**Table 6:** Effect of fermentation duration on feed milk value (FMV) in by-products from coffee bean processing based on net energy for lactation

Feed milk value (kg/kg feed)	Control (C)	Fermentation Duration (days) (F)				SEM	p-value	Contrast^1)^		
		
	F0	F7	F14	F21	F28			C vs F	L	Q
FMV	2.27	2.19	2.27	2.26	2.26	0.010	0.001	0.036	0.225	0.055

SEM, standard error of mean.

1) Polynomial contrast: L, linear; Q, quadratic.

**Table 7:** Effect of fermentation duration on molecular spectral characteristics using molecular spectroscopy -FTIR-ATR in by-products from coffee bean processing impacted by fermentation duration

Item^1)^	Wavenumber (cm^−1^)	Control (C)	Fermentation duration (days) (F)				SEM	p-value	Contrast^2)^		
		
		F0	F7	F14	F21	F28			C vs F	L	Q
Area											
STCHO	Ca. 1,488–1,184	31.49	31.73	31.95	32.56	31.90	0.447	0.565	0.303	0.272	0.420
CC	Ca. 1,300–1,184	4.360	4.246	4.502	4.668	4.566	0.121	0.187	0.340	0.054	0.889
NSCHO	Ca. 1,184–924	75.580	72.96	73.20	75.64	74.47	1.626	0.663	0.425	0.928	0.421
Heights											
Lignin	Ca. 1,507	0.001	0.000	−0.003	0.007	0.003	0.002	0.030	0.819	0.089	0.225
CH_bending	Ca. 1,460	0.136	0.147	0.134	0.118	0.130	0.008	0.276	0.695	0.158	0.973
STCHO_pk1	Ca. 1,418	0.146	0.148	0.147	0.150	0.146	0.002	0.818	0.566	0.791	0.490
STCHO_pk2	Ca. 1,397	0.157	0.162	0.158	0.152	0.158	0.003	0.438	0.933	0.410	0.924
STCHO_pk3	Ca. 1,320	0.123	0.121	0.127	0.135	0.129	0.003	0.092	0.155	0.027	0.566
CC	Ca. 1,237	0.076	0.076	0.077	0.080	0.079	0.001	0.355	0.387	0.092	0.956
HC	Ca. 1,150	0.131	0.119	0.120	0.121	0.115	0.003	0.154	0.023	0.041	0.474
NSCHO_pk1	Ca. 1,102	0.353	0.339	0.359	0.381	0.356	0.011	0.189	0.670	0.208	0.644
NSCHO_pk2	Ca. 1,078	0.453	0.423	0.438	0.459	0.440	0.127	0.374	0.376	0.833	0.566
NSCHO_pk3	Ca. 1,030	0.617	0.594	0.593	0.617	0.608	0.129	0.539	0.354	0.916	0.309

SEM, standard error of mean.

1) STCHO, structural carbohydrate (area at ca. 1,488 to 1,184 cm^−1^); CC, cellulosic compounds (area at ca. 1,300 to 1184 cm^−1^); NSHCO, non-structural carbohydrates (area at ca. 1,184 to 924 cm^−1^); Lignin, height at ca.1,507 cm^−1^; CH_bending, height at ca. 1,460 cm^−1^; STCHO_pk1, structural carbohydrate peak1 (height at ca. 1,418 cm^−1^); STCHO_pk2, structural carbohydrate peak2 (height at ca. 1,397 cm^−1^); STCHO_pk3, structural carbohydrate peak3 (height at ca. 1,320 cm^−1^); CC, cellulose (height at ca. 1,237 cm^−1^); HC, hemicellulose (height at ca. 1,150 cm^−1^); NSCHO_pk1, non-structural carbohydrate peak1 (height at ca. 1,102 cm^−1^); NSCHO_pk2, non-structural carbohydrate peak2 (height at ca. 1,078 cm^−1^); NSCHO_pk3, non-structural carbohydrate peak3 (height at ca. 1,030 cm^−1^).

2) Polynomial contrast: L, linear; Q, quadratic.

**Table 8:** Correlation between carbohydrate chemical profile and carbohydrate molecular structure spectra profiles in by-products from coffee bean processing impacted by microorganism fermentation duration

Item (% DM)	Carbohydrate molecular profiles^1)^									

	r^2)^									

	TC1H	TC2H	TC3H	TC4H	CECH	STC1H	STC2H	STC3H	STC4H	LigH
NDF	−0.22	−0.52	−0.50	−0.14	−0.04	−0.42	0.00	0.07	0.24	0.02
ADF	−0.40	−0.60^*^	−0.53^*^	−0.03	−0.23	−0.42	−0.11	−0.10	0.22	0.11
ADL	−0.17	−0.30	−0.54	0.46	−0.35	−0.80^**^	0.14	0.14	0.47	−0.15
Sugar	0.28	0.47	0.34	0.37	−0.08	0.01	−0.11	−0.10	−0.13	0.08
Starch	−0.16	−0.31	0.02	−0.06	0.19	−0.08	−0.15	−0.52	−0.06	0.64^*^

DM, dry matter; NDF, neutral detergent fibre; ADF, acid detergent fibre; ADL, acid detergent lignin.

1) TC1H, total carbohydrate peak 1 height; TC2H, total carbohydrate peak 2 height; TC3H, total carbohydrate peak 3 height; TC4H, total carbohydrate peak 4height; CECH, cellulosic compound height; STC1H, structural carbohydrate peak 1 height; STC2H, structural carbohydrate peak 2height; STC3H, structural carbohydrate peak 3 height; STC4H, structural carbohydrate peak 4 height; LigH, lignin height.

* p<0.05;

** p<0.001.

2) r, coefficient of correlation considering Spearman correlation.

**Table 9:** Correlation between energy values and carbohydrate molecular structure spectral profiles in by-products from coffee bean processing impacted by fermentation duration

Item^2)^	Carbohydrate molecular spectral profiles^1)^											
	r^3)^											
	TCA	TC1H	TC2H	TC3H	TC4H	CECA	STCA	STC1H	STC2H	STC3H	STC4H	LigH
tdNDF	−0.31	−0.26	−0.51	−0.31	−0.53	−0.11	−0.12	0.00	0.01	−0.10	0.08	0.05
tdNFC	0.32	0.29	0.50	0.37	0.38	0.29	0.27	0.15	−0.06	−0.03	−0.17	0.07
tdCP	−0.25	−0.21	−0.31	−0.31	−0.27	−0.27	−0.39	−0.15	0.02	0.05	0.06	−0.20
tdFA	0.25	0.27	0.29	0.33	−0.44	0.60^*^	0.25	0.58	−0.19	−0.09	−0.44	0.20
TDN_1x_	0.53	0.51	0.66^*^	0.62^*^	0.16	0.62^*^	0.41	0.42	−0.24	0.10	−0.40	0.10
DE_1x_	0.58^*^	0.56^*^	0.68^*^	0.64^*^	0.16	0.62^*^	0.23	0.38	−0.31	0.05	−0.50	0.09
DE_p3x_	0.59^*^	0.54^*^	0.71^**^	0.61^*^	0.14	0.66^*^	0.31	0.44	−0.15	0.11	−0.44	0.10
ME_p3x_	0.58^*^	0.53^*^	0.69	0.60^*^	0.13	0.66^*^	0.31	0.44	−0.15	0.11	−0.43	0.10
NE_m_	0.52	0.51	0.63	0.60^*^	0.14	0.59^*^	0.23	0.38	−0.36	0.02	−0.50	0.06
NE_g_	0.62^*^	0.59^*^	0.74	0.61^*^	0.19	0.60^*^	0.22	0.37	−0.17	0.11	−0.44	0.11
NE_Lp3x_	0.62^*^	0.59^*^	0.74	0.61^*^	0.19	0.60^*^	0.22	0.37	−0.17	0.11	−0.44	0.11

1) TCA, total carbohydrate area; TC1H, total carbohydrate peak 1 height; TC2H, total carbohydrate peak 2 height; TC3H, total carbohydrate peak 3 height; TC4H, total carbohydrate peak 4height; CECA, cellulosic compound area; STCA, structural carbohydrate area; STC1H, structural carbohydrate peak 1 height; STC2H, structural carbohydrate peak 2height; STC3H, structural carbohydrate peak 3 height; STC4H, structural carbohydrate peak 4 height; LigH, lignin height.

* p<0.05;

** p<0.001.

2) tdNDF, truly digestible neutral detergent fibre; tdNFC, truly digestible non-fibre carbohydrate; tdCP, truly digestible crude protein; tdFA, truly digestible fatty acid; TDN_1x_, total digestible nutrient at maintenance level; DE_1x_, digestible energy at maintenance level; ME_p3x_, metabolizable energy at production level at 3 times maintenance intake; NE_m_, net energy for maintenance; NE_g_, net energy for growth; NE_Lp3x_, net energy for lactation at production level at 3 times maintenance intake.

3) r, coefficient of correlation considering Spearman correlation.
